# Indigenous Ethiopian okra (*Abelmoschus esculentus)* mucilage: A novel ingredient with functional and antioxidant properties

**DOI:** 10.1002/fsn3.596

**Published:** 2018-02-02

**Authors:** Habtamu Fekadu Gemede, Gulelat Desse Haki, Fekadu Beyene, Sudip Kumar Rakshit, Ashagrie Z. Woldegiorgis

**Affiliations:** ^1^ Department of Food Technology and Process Engineering Wollega University Nekemte Ethiopia; ^2^ Department of Food Science and Technology Botswana Collage of Natural Science and Agriculture Botswana University Botswana; ^3^ Department of Chemical Engineering Canada Research Chair (Tier 1) Lakehead University Thunder bay ON Canada; ^4^ Center for Food Science and Nutrition program Addis Ababa University Addis Ababa Ethiopia

**Keywords:** accessions, antioxidant, functional, mucilage, Okra, pods

## Abstract

Functional and antioxidant properties of mucilage extracted from the pods of eight okra accessions grown in Benishangul‐Gumuz region, Western Ethiopia, were evaluated. This study had shown that the mucilage contents of the pods of eight okra accessions ranged from 1.25 to 3.45 g/100 g. Functional properties of the mucilage of okra pods varied significantly (*p* < .05) and had respective ranges of bulk density of 0.58–0.64 g/ml; water absorption capacity of 2.45–4.60 ml/g; oil absorption capacity of 0.02–3.64 ml/g; emulsifying capacity of 42.22%–74.45%; emulsion stability of 42.22%–74.45%; foaming capacity of 50.51%–62.50%, and foam stability of 36.04%–54.35%. Total phenolic and flavonoid contents of the mucilage of the pods of okra accessions ranged from 4.66 to 49.93 mg GAE/g and 8.18–18.72 mg CE/g, respectively. The effective concentration (EC_50_) values (mg/ml) of mucilage of okra pods varied from 3.15 to 6.60 and 1.10 to 1.85 for DPPH scavenging and metal‐chelating activity, respectively. The study revealed that the mucilage of the pods of okra accessions was found to exhibit good functional properties and can offer a great potential in various food systems. Particularly, mucilage of the pods from OPA#5 and OPA#7 had desirable water and oil absorption capacities, whereas the mucilage of accession OPA#1 and OPA#6 had high emulsifying and foaming properties. The results also demonstrated that okra pod mucilage had potential sources of natural antioxidant.

## 1 INTRODUCTION

Mucilage is a plant hydrocolloid which is a polymer of a monosaccharide or mixed monosaccharide (Deogade, Deshmukh, & Sakarkar, 2012). It is a common constituent of plants such as okra (*Abelmoschus esculentus*), psyllium (*Plantago species*), yellow mustard (*Sinapis alba*), and flaxseed (*Linum usitatissimum*) (Kaewmanee et al., 2014). Okra (*Abelmoschus esculentus*) pod is an important vegetable in the tropics and subtropics (Jideani & Bello, 2009), and it contains mucilage which is thick slimy polysaccharides and is used to thicken soups and stews (Ahiakpa et al., 2014; Biswal, Karna, & Patel, 2014).

Okra typically differs from most other common vegetables in having high mucilage content (Jideani & Bello, 2009). Okra mucilage has the potential for use as food, nonfood products, and medicine (Haruna, Aliyu, & Bala, 2016; Kumar et al., 2010). The food applications include a whipping agent for reconstituted egg whites, an additive in the formulation of flour‐based adhesives, an additive for clarifying sugarcane juice. It is also used to modify the food quality in terms of food stability, texture, and appearance properties by acting as an emulsifier, thickener, gelling agent, or texture modifier (Noorlaila, Siti Aziah, Asmeda, & Norizzah, 2015). Okra mucilage also contributes to improved functionality, especially water‐binding, emulsifying, and foaming properties of food products (Jideani & Bello, 2009).

Mucilage from okra contains significant levels of protein, carbohydrate, neutral sugars, minerals, and other complex polysaccharides (Ahiakpa et al., 2014). It is medically proven to be linked to anticancer, antimicrobial, hypoglycemic, anti‐ulcer activities (Ansari, Houlihan, Hussain, & Pieroni, 2005), as well as its ability to bind cholesterol and bile acid carrying toxins by filtering the liver (Shui & Peng, 2004). The chemical compositions, molecular structures, monosaccharide sequences, glycoside linkages configuration, and position in the backbone and side chains are some of the factors that can affect the functional properties of natural plant mucilage (Mirhosseini & Amid, 2012).

There are few studies on the functional properties of mucilage from okra vegetables (Adetuyi & Dada, 2014; Jideani & Bello, 2009; Noorlaila et al., 2015; Woolfe, Chaplin, & Otchere, 1977). However, there is very little information reported on the antioxidant properties of okra mucilage. Despite the fact that Ethiopia is native to okra vegetable, there is no published literature on functional and antioxidant properties of mucilage of Ethiopian okra vegetable. Hence, the objectives of this study were to determine yield composition, and functional and antioxidant properties of mucilage extracted from pods of eight Indigenous Ethiopian okra accessions in order to evaluate its potentials and applications in food uses.

## 2 MATERIALS AND METHODS

### 2.1 Sample collection

Okra accessions were collected from different agro‐ecological locations in the regions by Assosa Agricultural Research Center in 2012 and 2013 harvesting seasons and planted on the research center plot under similar agronomic practice and management conditions during the 2014 main cropping season. The pods of eight okra accessions, namely OPA#1, OPA#2, OPA#3, OPA#4, OPA#5, OPA#6, OPA#7, and OPA#8, were collected from the center plots during the 2014 main okra harvesting season.

### 2.2 Mucilage extraction

The mucilage of the pods of okra accessions was extracted according to the procedure described by Farooq, Malviya, and Sharma (2013). About 100 g of the sliced and dried okra was dissolved in 300 ml of distilled water. It was heated in a water bath with continuous stirring for 1 hr at 60°C. The concentrated solution was filtrated through a muslin cloth and cooled to room temperature. About 20 ml of acetone was added to the concentrated solution. The mucilage was filtered again through a muslin cloth and cooled. The filtered mucilage was further dried to constant weight at 45°C in drying oven. Hard mucilage cake was ground into a fine powder by mortar and pestle until it is small enough to pass through 0.425‐mm sieve size. The mucilage powder was packed in airtight polyethylene plastic bags and was stored in a desiccator until required for analysis.

### 2.3 Determination of functional properties

#### 2.3.1 Determination of bulk density

The bulk density of the mucilage powder was determined according to the method described by Gupta, Lakshmi, Manjunath, and Prakash (2005). About two grams of mucilage powder was placed in 10‐ml test tube. The test tube was tapped several times (minimum 10 times) on the laboratory bench to compact the mucilage powder. The final bulk volume was recorded.

#### 2.3.2 Determination of water and oil absorption capacity

Water and oil absorption capacities were determined according to the method described by Aremu, Olaofe, and Akintayo (2007). One gram of sample was mixed with 10 ml of distilled water or oil (specific gravity of 0.929) in a centrifuge tube and allowed to stand at room temperature for 1 hr. It was then centrifuged at 200 rpm for 30 min, and the supernatant was transferred into a 10‐ml graduated cylinder.

#### 2.3.3 Determination of emulsifying properties

The emulsifying capacity and emulsion stability of the mucilage of the pods of okra accessions were determined according to the method described by Thanatcha and Pranee (2011). About 1 g of mucilage powder was dissolved in 50 ml of distilled water, and 50 ml refined oil was added into the mixture. Then, the mixture was homogenized for 1 min and was centrifuged at 2,000 rpm for 5 min. Finally, the height of emulsified layer was measured and compared with the height of the whole layer. The emulsion stability was estimated after heating the emulsion contained in calibrated centrifuged tube at 80°C for 3 min in a water bath. The heated emulsion was cooled for 15 min under running tap water and was centrifuged for 15 min.

#### 2.3.4 Determination of foaming properties

Foaming properties including foaming capacity and stability of the flour samples were determined according to Aremu et al. (2007). One gram of the sample was dispersed in 50 ml of distilled water. The resulting solution was vigorously whipped for 30 min in a kenwood blender and poured into a 100‐ml graduated cylinder. The volume before and after whipping was recorded, and the foaming capacity was calculated as percentage volume increase. Foaming stability was determined as the volume of foam that was remained after 8 hr and was expressed as a percentage of the initial volume**.**

#### 2.3.5 Determination of antioxidant properties

##### Methanolic extraction

Methanolic extraction of mucilage was conducted according to the procedures outlined by Woldegiorgis, Abate, Haki, and Ziegler (2014). About 10 g of sample was extracted by stirring with 100 ml of methanol at 25°C at 150 rpm for 24 hr using temperature shaker incubator (ZHWY‐ 103B) and then filtered through Whatman No. 4 paper. The residue was extracted again with additional 100 ml portions of methanol as described above. The combined methanolic extracts were evaporated to dryness at 40°C using rotary evaporator (Stuart R3300), and redissolved in methanol at the concentration of 50 mg/ml and stored at 4°C for further use.

#### 2.3.6 Determination of total phenolics

Total phenolic content (TPC) was determined based on the procedures described by Ferreira, Baptista, Vilas‐Boas, and Barros (2007), using gallic acid as a standard for the calibration curve. One milliliter of the sample was mixed with 1 ml of Folin–Ciocalteu's phenol reagent. After 3 min, 1 ml of saturated sodium carbonate (20%) solution was added to the mixture and adjusted to 10 ml of distilled water. The reaction was kept in the dark for 90 min after which the absorbance was measured spectrophotometrically at 725 nm using a UV‐VIS Spectrophotometer (Agilent Cary Corporation, 1001, Kyoto, Japan). Gallic acid was used to construct the calibration curve. The concentration of the standard solution ranged from 0.5 to 100 μg/ml (absorbance = 427.63 gallic acid μg + 0.1453, *R* ^2^ = .999).

#### 2.3.7 Determination of total flavonoids

Total flavonoid content (TFC) was determined by a colorimetric method as described by Xu and Chang (2007) and Woldegiorgis et al. (2014). About 0.25 ml of the extract was mixed with 1.25 ml of deionized water and 75 μl of a 5% NaNO_2_ solution. After 6 min, 150 μl of a 10% AlCl_3_.6H_2_O solution was added to the mixture. The mixture was incubated at room temperature for 5 min, after which 0.5 ml of 1 M NaOH and 2.5 ml of deionized water were added. The mixture was then thoroughly vortexed for 5 min, and the absorbance of the pink color was measured at 510 nm against the blank. For calibration curve (+), catechin was used with a concentration range of 10–1000 μg/ml (absorbance = 27.05 catechin μg + 0.1453, *R* ^2^ = .995).

#### 2.3.8 Determination of DPPH scavenging activity

DPPH *(2,2′‐diphenyl‐1 ‐ picrylhydrazyl)* scavenging activity of the methanolic extract of the sample was determined according to the procedure proposed by Woldegiorgis et al. (2014). A 0.004% solution of DPPH radical solution in methanol was prepared, and then, 4 ml of this solution was mixed with 1 ml of various concentrations (2–14 mg/ml) of the extracts in methanol. Finally, the samples were incubated for 30 min in the dark at room temperature. Scavenging capacity was read spectrophotometrically (Perkin Elmer Lamda 950 UV/Vis/NIR) by monitoring the decrease in absorbance at 517 nm. The absorption maximum was first verified by scanning freshly prepared DPPH from 200 to 800 nm using the scan mode of the spectrophotometer. Butyl hydroxytoluene (BHT) and L‐ascorbic acid were used as the positive control with the same concentrations as above. The extract concentration providing 50% of radicals scavenging activity (EC_50_) was calculated from the graph of DPPH scavenging activity percentage against extract concentration.

#### 2.3.9 Determination of metal‐chelating effects

Metal‐chelating effects on ferrous ions were determined according to Woldegiorgis et al. (2014). About 2 ml of various concentrations (0.05–1.5 mg/ml) of the extracts in methanol was added to a solution of 2 mmol/L FeCl_2_ (0.05 ml). The reaction was initiated by the addition of 5 mmol/L ferrozine (0.2 ml). Total volume was adjusted to 5 ml with methanol, and then, the mixture was shaken vigorously and left at room temperature for 10 min. The absorbance of the solution was measured at 562 nm. A lower absorbance indicates a higher ferrous ion‐chelating capacity, and 2, 2‐bipyridyl, disodium ethylenediaminetetracetate (EDTA) was used as a positive control. The extract concentration providing 50% inhibition (EC_50_) was calculated from the graph of ferrous ion inhibition percentage against extract concentration. The inhibition percentage of ferrozine‐ Fe^2+^ complex formation was then calculated.

### 2.4 Statistical analysis

The completely randomized design was used with two replicates. All the statistical analyses were performed on the results obtained using SPSS version 20.0 for windows. One‐way analysis of variance was used to evaluate the data. Means of the experiment were separated by Duncan's multiple range test and reported as a mean ±  standard error. A *p*‐value of .05 or less was considered as statistically significant. Graphs of effective concentration at 50% (EC_50_) of the respective antioxidant activities were constructed using Microsoft Excel.

## 3 RESULTS AND DISCUSSION

### 3.1 Mucilage yields

**Figure 1:**
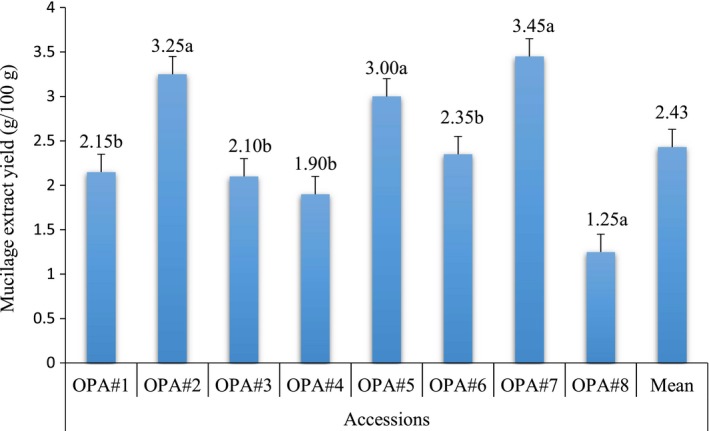
Mucilage yields from pods of eight okra accessions

The mucilage yield from pods of eight different okra accessions is presented in Figure 1. The mucilage yield of the pods of okra accessions was ranged from 1.25 to 3.45 g/100 g. The mucilage yield of OPA#7 accession was significantly (*p* < .05) high, whereas it was low in OPA#8 accession on a dry‐weight basis. The variation in mucilage yield was attributed to species type, maturity at harvesting time, effect of drying, genetic factor, the season of collection, and topographic variation such as rain distribution, temperature, and soil type. (Gebresamuel & Gebre‐Mariam, 2011). Moreover, Kaewmanee et al. (2014) pointed out that the extraction yield of mucilage can vary as a function of environmental factors, such as the climatic condition and crop age.

The mean mucilage content (2.43 g/100 g) of Ethiopian okra is higher than the value reported by Adetuyi and Dada (2014) for Nigerian okra (1.4 g/100 g). The mean mucilage yield (2.43 g/100 g) of Ethiopian okra is also higher than the yields of mucilage (1.10%–2.55%) from leaves of rose cactus reported by Hong and Ibrahim (2012). The mucilage yield of linseed (3.6%–9.4%) reported by Fedeniuk and Biliaderis (1994) is also higher than the present finding. Kaewmanee et al. (2014) also reported the mucilage yield flaxseed (*Linumusitatissimum*) cultivar ranges from 1.80 to 6.65%.

### 3.2 Functional properties

#### 3.2.1 Bulk density

Table 1 shows the bulk density of mucilage of the pods of eight okra accessions. The bulk density of the mucilage flour ranged from 0.58 to 0.64 g/ml. Bulk density of the mucilage flour of OPA#6 accession was highest (0.64 g/ml) but this was not significantly (*p* > .05) different from OPA#3, OPA#8, and OPA#4 accessions, whereas OPA#2 was the lowest (0.58 g/ml) but this was not significantly (*p* > .05) different from the rest of accessions except OPA#6. The mean bulk density (0.60 g/ml) of the mucilage in this finding was lower than the value (0.69 g/ml) reported by Farooq et al. (2013) for okra mucilage, which is stated as heavy in nature. The bulk density in the present finding is also lower than the value (0.68–0.69 g/ml) reported by Gebresamuel and Gebre‐Mariam (2011) for cactus pears (*Opuntia* spp.) grown in northern Ethiopia.

**Table 1:** Functional properties of mucilage from pods of okra accessions

Accessions	BD (g/ml)	WAC (ml/g)	OAC (ml/g)	EC (%)	ES (%)	FC (%)	FS (%)
OPA#1	0.59 ± 0.02^b^	2.70 ± 0.30 ^cd^	2.02 ± 0.11^e^	64.39 ± 3.06^abc^	59.20 ± 2.34^ab^	60.41 ± 3.72^ab^	53.56 ± 0.99^a^
OPA#2	0.58 ± 0.02^b^	3.70 ± 0.10^b^	2.98 ± 0.24^bc^	42.22 ± 3.24^e^	39.83 ± 2.54^c^	52.80 ± 0.27^ab^	36.04 ± 0.69^e^
OPA#3	0.61 ± 0.01^ab^	3.05 ± 0.15^c^	2.38 ± 0.06^de^	48.33 ± 6.22^de^	42.94 ± 4.34^c^	50.51 ± 0.50^b^	40.40 ± 1.01^de^
OPA#4	0.61 ± 0.02^ab^	3.10 ± 0.10^c^	2.52 ± 0.10^d^	64.28 ± 2.40^abc^	57.87 ± 0.47^ab^	58.20 ± 0.90^ab^	46.32 ± 0.87^b^
OPA#5	0.59 ± 0.01^b^	4.40 ± 0.10^a^	3.64 ± 0.11^a^	60.33 ± 2.64^bc^	54.71 ± 0.86^b^	52.39 ± 0.81^ab^	47.09 ± 0.28^b^
OPA#6	0.64 ± 0.01^a^	2.45 ± 0.05^d^	2.08 ± 0.17^e^	74.45 ± 2.82^a^	65.26 ± 2.93^a^	62.50 ± 0.47^a^	54.35 ± 2.45^a^
OPA#7	0.58 ± 0.01^b^	4.05 ± 0.25^ab^	3.28 ± 0.04^ab^	55.35 ± 2.66^cd^	53.27 ± 3.27^b^	54.41 ± 0.80^ab^	45.61 ± 1.28^bc^
OPA#8	0.61 ± 0.01^ab^	4.60 ± 0.20^a^	2.68 ± 0.07^cd^	68.01 ± 2.43^ab^	56.70 ± 0.45^ab^	54.69 ± 2.61^ab^	41.18 ± 2.64^cd^
Mean	0.60	3.51	2.69	59.67	53.72	55.74	45.57

BD, bulk density; WAC, water absorption capacity; OAC, oil absorption capacity; EC, emulsifying capacity; ES, emulsion stability; FC, foaming capacity; FS, foam stability.

Means not followed by the same superscript letters in each column of the pod and seed are significantly (*p* < .05) different. Data are expressed as mean ± standard error of replicate determinations (*n* = 2).

#### 3.2.2 Water absorption capacity

The result of water absorption capacity of the mucilage of the pods of eight okra accessions is given in Table 1. Water absorption capacity of the pod mucilage of okra accessions was ranged from 2.45 to 4.60 ml/g. Water absorption capacity of OPA#8 was the highest but this was not significantly (*p* > .05) different from OPA#5 and OPA#7 accessions. It was low in OPA#1 and OPA#2. The mean water absorption capacity of mucilage from pods of okra accessions in this study is lower than the water absorption capacity reported by Hong and Ibrahim (2012) for rose cactus mucilage (461.87%) but higher than Arabic gum (17.13%). The ability of mucilage to hold water‐producing gels or highly viscous solution is desirable in industrial application (Simas‐Tosin et al., 2010).

#### 3.2.3 Oil absorption capacity

The oil absorption capacity of the mucilage of the pods of eight okra accessions is presented in table 6.1. The oil absorption capacity of the mucilage of the pods of okra accessions ranged from 2.02 to 3.64 ml/g. The oil absorption capacity of the pods of okra accession OPA#5 was the highest but this as not significantly (*p* > .05) different from OPA#7. On the other hand, the oil absorption capacity of OPA#1 and OPA#6 was the lowest but this also did not significantly (*p* > .05) different from OPA#3. Oil absorption capacity is great importance, as fat acts as flavor retainer and also increases soft texture to mouthfeel of foods, especially bread and other baked foods (Akobundu, 2009; Aremu, Olonisakin, Atolaye, & Ogbu, 2006). A high oil absorption capacity is valuable in ground meat formulations, meat replacers and extenders, doughnuts, pancakes, and baked foods (Amandikwa & Chinyere, 2012).

#### 3.2.4 Emulsifying capacity

The result of the emulsifying capacity of the mucilage of the pods of okra accessions is presented in Table 1. The emulsifying capacity of the pod mucilage of okra accessions ranged from 42.22 to 74.45%. The emulsifying capacity of OPA#2 was high but did not significantly (*p* > .05) different from OPA#3 accession. It was low in OPA#6 but this also did not significantly (*p* > .05) different OPA#8, OPA#1, and OPA#4 accessions. Protein being the surface active agents can form and stabilize the emulsion by creating electrostatic repulsion on oil droplet surface (Kaushal, Kumar, & Sharma, 2012). The capacity of proteins to enhance the formation and stabilization of emulsion is critical for many applications in food products such as chopped and comminuted meat, cake batter, coffee whitener, milk, mayonnaise, salad dressing, and frozen dessert (Elbaloula, Runqiang, Qianghui, & Zhenxin, 2014). The mucilage of all the accessions showed relatively good emulsion capacities.

#### 3.2.5 Emulsion stability

Emulsion stability of the mucilage of the pods of okra accessions is shown in Table 1. Emulsion stability of the pod mucilage ranged from 42.22 to 74.45%. Emulsion stability of OPA#2 was high but did not significantly (*p* > .05) different from OPA#3 accession. It was low in OPA#6 but this also did not significantly (*p* > .05) different OPA#8, OPA#1, and OPA#4 accessions. All the mucilage samples showed a high stability of emulsion lasting 60 min after whipping. The mean emulsion stability of okra mucilage in the present study is lower than the value (80%) reported by Kaewmanee et al. (2014) for flax cultivars. The mucilage of the pods of okra accessions showed relatively good emulsion stability. Capitani, Tomás, and Nolasco (2013) also reported that regarding the formulation of stable emulsions, the meal with mucilage is recommended for use given the role of mucilage as a thickening agent.

#### 3.2.6 Foaming capacity

Foaming capacity of mucilage of the pods of okra accessions is presented in Table 1. Foaming capacity of the pod mucilage ranged from 50.51 to 62.50%. Foaming capacity of OPA#6 was significantly (*p* < .05) high, and it was low in OPA#3 but the rest of accessions did not significantly (*p* > .05) different from OPA#6 and OPA#3. The mean foaming capacity (55.74%) of okra mucilage in the present finding is higher than the value (20%–25%) reported by Kaewmanee et al. (2014) for the mucilage of flax cultivars.

#### 3.2.7 Foam stability

Foam stability of mucilage of the pod accessions is shown in table 6.1. Foaming capacity of the pod mucilage ranged from 36.04 to 54.35%. Foaming capacity of OPA#6 and OPA#1 was significantly (*p* < .05) high, and it was low in OPA#2 but this did not significantly (*p* > .05) different from OPA#3 accession. The ability to form stable foam is an important property in whipped toppings, frozen desserts, and sponge cakes (Adelakun, Ade‐Omowaye, Adeyemi, & Van de Venter, 2012); thus, the mucilage of the pods of okra accessions particularly OPA#6 and OPA#1 accessions is the best candidate for the food that requires high stable foam.

### 3.3 Total phenolics and flavonoids

#### 3.3.1 Total phenolics

**Figure 2:**
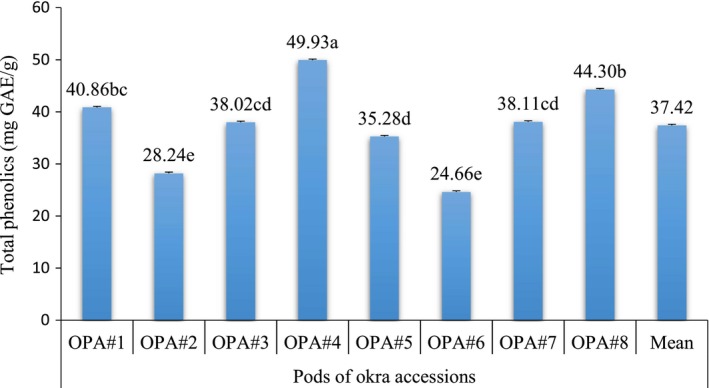
Total phenolics (mg GAE/g) of mucilage from pods of eight okra accessions

The result of the total phenolic contents of the mucilage of the pods of eight okra accessions is shown in Figure 2. Total phenolic contents of the pods of okra mucilage ranged from 24.66 to 49.93 mg GAE/g. The pods of accession OPA#4 were significantly (*p* < .05) high, and it was low in OPA#6 and OPA#2. The mean total phenolic content of okra mucilage was 37.42%. Plants rich in phenolics are being used in the food industry because they retard oxidative degradation of lipids and improve the quality and nutritional value of food (Saeed, Khan, & Shabbir, 2012).

#### 3.3.2 Total flavonoids

**Figure 3:**
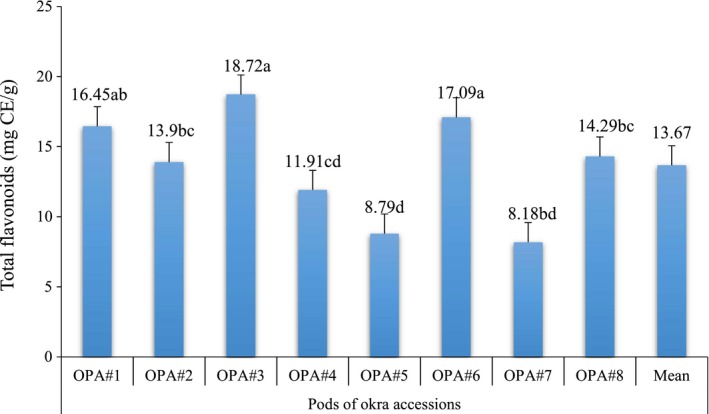
Total flavonoids (mg CE/g) of mucilage from pods of eight okra accessions

The result of total flavonoid contents of the mucilage of the pods of eight okra accessions is presented in Figure 3. Total phenolic contents of the pods of eight okra mucilage ranged from 8.18 to 18.72 mg CE/g. The pods of OPA#3 accession were high but did not significantly (*p* > .05) different from OPA#6 and OPA#1. It was low in OPA#7 but this also did not significantly (*p* > .05) different from OPA#5 and OPA#4. The mean total flavonoid content of okra mucilage was 13.67 mg CE/g. Flavonoids are highly effective scavengers of most oxidizing molecules, including singlet oxygen, and various other free radicals implicated in several diseases (Saeed et al., 2012).

### 3.4 Antioxidant activity assays

#### 3.4.1 DPPH scavenging activity

**Figure 4:**
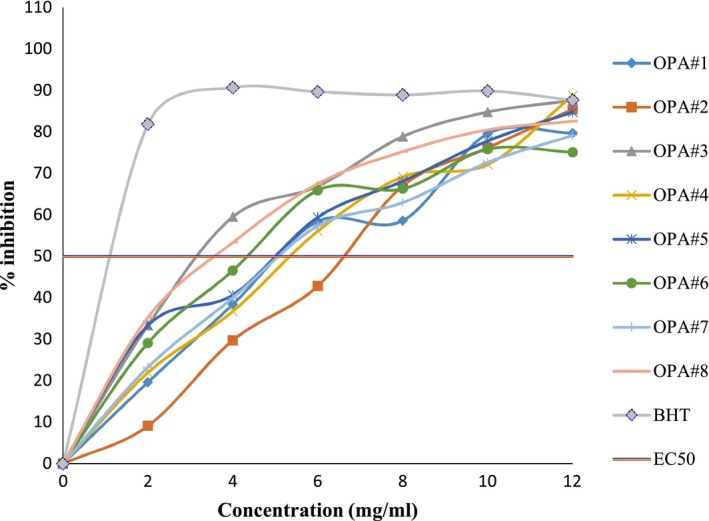
DPPH scavenging activity of mucilage from okra pod accessions and control

The result of concentration‐response curves of DPPH scavenging activity of the mucilage of the pods of eight okra extracts with positive controls is shown in Figure 4. The synthetic antioxidant of Butylated hydroxytoluene (BHT) was used as a positive control using the same concentration. The percentage inhibition of DPPH scavenging activities of mucilage of the pods of eight okra accessions was evaluated at concentrations of 2–12 mg/ml. There was an increase in DPPH radical scavenging activity with increasing concentration of mucilage of the pods extract used in this study. This result agreed with the report of Motalleb, Hanachi, Kua, Fauziah, and Asmah (2005), who showed that the scavenging effects on the DPPH radical increase sharply with increasing concentration of the samples and standards. At each concentration, the percentage inhibition of the mucilage of OPA#3 and OPA#8 accessions was relatively higher than the rest of accessions. The scavenging effect of synthetic antioxidant BHT was higher than all the mucilage of the pod accessions.

#### 3.4.2 Metal‐chelating effect

**Figure 5:**
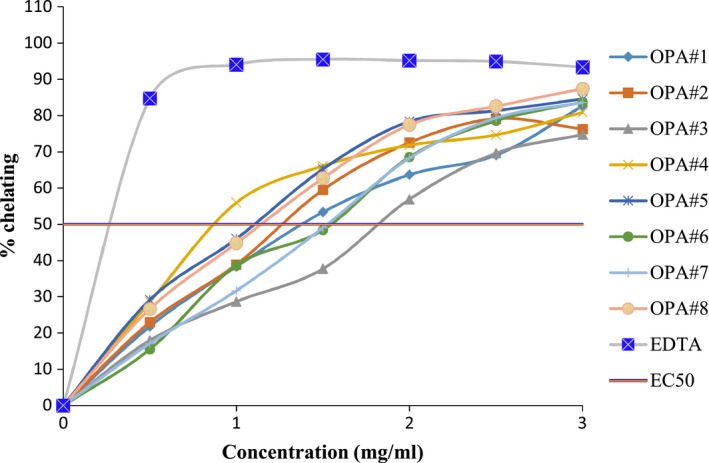
Metal‐chelating effect of mucilage from okra pod accessions and control

The metal‐chelating effect of the mucilage of the pods of okra accessions with a positive control (EDTA) is shown in Figure 5 The concentration range chosen to compare chelating power and to interpolate the EC_50_ (effective concentration at which 50% Fe^2+/^ferrozine complex is inhibited) is from 0.5 to 3 mg/ml. At each concentration, the chelating effects of the mucilage of OPA#4, OPA#5, and OPA#8 accessions were relatively higher, and OPA#3 was lower than the rest of accessions. The chelating effect of synthetic antioxidant EDTA was higher than all the mucilage of the pod accessions.

#### 3.4.3 Effective concentration values

The effective concentrations (EC_50_) values for DPPH scavenging and metal‐chelating effects of mucilage of the pods of okra accessions with positive controls are shown in Table 2. The EC_50_ values (mg/ml) of mucilage of okra pod accessions ranged from 3.15 to 6.60 and 1.10 to 1.85 for DPPH scavenging and metal‐chelating effect, respectively.

**Table 2:** Effective concentration (EC_50_) values (mg/ml) of mucilage from okra pod accessions

Accessions	DPPH scavenging (EC_50_ a)	Chelating effect (EC_50_ b)
OPA#1	5.05	1.38
OPA#2	6.60	1.27
OPA#3	3.15	1.85
OPA#4	5.28	1.10
OPA#5	5.00	1.15
OPA#6	4.35	1.60
OPA#7	5.10	1.55
OPA#8	3.55	1.20
Mean	4.76	1.39
BHT	1.10	—
EDTA	—	0.87

a EC_50_ (mg/ml): effective concentration at which 50% of DPPH radicals are scavenged.

b EC_50_ (mg/ml): effective concentration at which 50% Fe^2+^/ferrozine complex are inhibited.

The result of this study revealed that the mucilage of the pods of accession OPA#3 and OPA#8 had better antioxidant properties with low EC_50_ values for DPPH scavenging and OPA#2 had low antioxidant properties with high EC_50_ values. The mucilage of the pods of accession OPA#4 and OPA#5 had a relatively high metal‐chelating effect. The synthetic antioxidant (BHT and EDTA), which was used as a positive control, had a superior performance with the least EC_50_ in all the assays, which indicate that the pod accessions of okra mucilage had low antioxidant activities.

## 4 CONCLUSION

Mucilage of the pods of eight okra accessions was extracted and characterized for their functional and antioxidant properties. From this study, it was revealed that pods of okra accessions contain a desirable amount of mucilage contents and are potential sources of natural antioxidants. The study also revealed that the mucilage of the pods of okra accessions was found to exhibit good functional properties and can offer a great potential in various food systems. Particularly, mucilage of the pods of OPA#5 and OPA#7 had desirable water and oil absorption capacities, whereas mucilage of accession OPA#1 and OPA6 had high emulsifying and foaming properties. In order to encourage the use of mucilage from pods of okra accessions, more research has to be conducted on its extraction optimization.

## CONFLICT OF INTEREST

None declared.
